# HSV-1 and Alzheimer’s disease: more than a hypothesis

**DOI:** 10.3389/fphar.2014.00097

**Published:** 2014-05-07

**Authors:** Roberto Piacentini, Giovanna De Chiara, Domenica D. Li Puma, Cristian Ripoli, Maria E. Marcocci, Enrico Garaci, Anna T. Palamara, Claudio Grassi

**Affiliations:** ^1^Institute of Human Physiology, Medical School, Università Cattolica del Sacro CuoreRome, Italy; ^2^Institute of Translational Pharmacology, National Research CouncilRome, Italy; ^3^Department of Public Health and Infectious Diseases, Sapienza University of RomeRome, Italy; ^4^San Raffaele Pisana Scientific Institute for Research, Hospitalization and Health Care, Telematic UniversityRome, Italy; ^5^Department of Public Health and Infectious Diseases, Institute Pasteur Cenci Bolognetti Foundation, Sapienza University of RomeRome, Italy; ^6^San Raffaele Pisana Scientific Institute for Research, Hospitalization and Health CareRome, Italy

**Keywords:** HSV-1, Alzheimer’s disease, recurrent infection, γ secretase, amyloid-β protein

## Abstract

Among the multiple factors concurring to Alzheimer’s disease (AD) pathogenesis, greater attention should be devoted to the role played by infectious agents. Growing epidemiological and experimental evidence suggests that recurrent herpes simplex virus type-1 (HSV-1) infection is a risk factor for AD although the underlying molecular and functional mechanisms have not been fully elucidated yet. Here, we review literature suggesting the involvement of HSV-1 infection in AD also briefly mentioning possible pharmacological implications of these findings.

## ALZHEIMER’S DISEASE, APP PROCESSING AND AMYLOID-β PRODUCTION/ACCUMULATION

Alzheimer’s disease (AD) is a neurodegenerative disorder characterized by progressive decline in cognitive functions leading to memory loss and dementia. It involves degeneration of limbic and cortical brain structures, especially in the temporal lobe. In 2010, it was estimated that 35.5 million individuals were affected by dementia in the world with a prediction that this number will increase to 65.7 million by 2030, and 115.4 million by 2050. The World Alzheimer Report 2010 (AD International) estimated that the total costs of dementia in 2010 were US$ 604 billions. Most (>90%) AD cases are sporadic. Only a minority of them have a genetic inheritance and are referred to as familial AD. The main risk factor for AD is aging. The disease afflicts 10% of the population over the age of 65 and 50% of the population over the age of 85. Several factors may increase the chances to develop AD (Imtiaz et al., 2014). Among the “genetic” risk factors, the carriage of a type 4 allele of the apolipoprotein E gene (APOE-ε4) has been widely recognized (see references in Kim et al., 2009). Among the “environmental” risk factors, persistent brain infections, particularly those induced by herpes simplex virus type-1 (HSV-1), seem to play a key role in AD pathogenesis. In a multifactorial disease like AD, to identify the contribution made by each factor and the underlying mechanisms is critical to the development of new therapeutic strategies to prevent the disease and/or slow down its progression.

From the neuropathological point of view, the brains of AD sufferers are characterized by the presence of two major hallmarks mainly located in the hippocampus and cortex: extracellular amyloid plaques composed of insoluble aggregates of the amyloid-β protein (Aβ) and intraneuronal neurofibrillary tangles, composed of hyperphosphorylated Tau protein (Kosik et al., 1986). Numerous other conditions, among which oxidative stress (Mattson et al., 1993) and inflammation, due to microglia activation and astrocytosis, may concur to produce the structural and functional alterations typically found in AD brain (Kitazawa et al., 2004).

Amyloid-β protein is a small protein (generally composed of 40 to 43 amino acids) generated by the proteolytic cleavage of the amyloid precursor protein (APP), a ubiquitous transmembrane protein whose functional role in cell biology has been only partially discovered. APP seems to be involved in neurite outgrowth and synaptogenesis, neuronal protein trafficking along the axon, transmembrane signal transduction, cell adhesion and Ca^2^^+^ signaling (Zheng and Koo, 2006; Octave et al., 2013). Among the various isoforms of APP, due to alternative splicing of *APP* gene located on chromosome 21 in humans, those of 695-amino acid length is prevalent in neurons whereas the other two forms, APP751 and APP770, are expressed in other tissues (Zhang et al., 2011).

Amyloid precursor protein is normally subjected to processing by specific enzymes called “secretases” (Nunan and Small, 2000). It may be processed along two different pathways. The first one, that is most frequent and “non amyloidogenic”, involves sequential proteolytic cleavages by the α- and γ-secretases. The former cuts APP at a position 83 amino acids from the C-terminus, thus producing a large N-terminal domain (sAPPα), normally secreted into the extracellular medium, whose function is not well defined. On the opposite site, the other 83-amino-acid C-terminal fragment (C83) is retained in the membrane and it is subsequently cleaved by the γ-secretase complex (that consists of presenilins, nicastrin, anterior pharynx-defective 1 and presenilin enhancer 2), producing a short fragment that is generally considered non-toxic and known as “p3”. The amyloidogenic pathway begins when APP undergoes cleavage by the β-secretase, also known as β-site APP cleaving enzyme 1 or BACE-1 (Vassar et al., 1999). β-secretase cuts APP 16 amino acids before α-secretase and yields two species, the large N-terminal ectodomain of the precursor and the 99-amino acid C-terminus stub (C99). Subsequent cleavage of the latter fragment by γ-secretase results in the formation of Aβ species containing 40 to 42 amino acids. This also means that APP cleavage by α-secretase prevents the Aβ formation. On the other C-terminal side of APP, γ-secretase (in both amyloidogenic and non-amyloidogenic pathways) also generate the “APP intracellular domain” (AICD), that has been reported to modulate the transcription of several genes (including APP itself, BACE-1 and the Aβ-degrading enzyme neprilysin), to regulate apoptosis and contribute to AD pathogenesis (Octave et al., 2013). In APP, the amino acid threonine in position 668 (Thr668) is an important site for its processing. Indeed, APP phosphorylation at Thr668 resulted in increased Aβ formation (Lee et al., 2003; Pierrot et al., 2006). This amino acid is on the C-terminal region of APP and its phosphorylation also regulates the activity of AICD. In fact, when AICD is phosphorylated at Thr668 it interacts with the Fe65 adapter protein (Bórquez and González-Billault, 2012) and enters the nucleus, where it may regulate gene transcription and induce neurodegeneration (Chang et al., 2006).

Amyloidogenic cleavage of APP is not confined to cell membrane; it also takes place in several cellular compartments (Vetrivel and Thinakaran, 2006), including the intermediate compartment of the endoplasmic reticulum (Cook et al., 1997), the trans-Golgi network (Choy et al., 2012), and the endosomal/lysosomal system, where APP processing is regulated by cytoplasmic phosphorylation at Thr668 (Lee et al., 2003). For these reasons, Aβ species may be secreted directly into the extracellular space, where their subsequent aggregation promotes senile plaque formation. Alternatively, it may remain within the cell or may be re-internalized in neurons, where it accumulates (Mohamed and Posse de Chaves, 2011). Some recent findings suggest that Aβ accumulating in neurons, particularly in the form of small oligomers (especially dimers and trimers) is the major determinant of the synaptic damage that highly correlates with the cognitive deficits characterizing the early phases of the disease preceding neuronal death (Tampellini et al., 2007; Mucke and Selkoe, 2012; Ripoli et al., 2013). However, Aβ is not a mere toxic peptide. It is constitutively produced and secreted by cells and, especially when present at very low concentration (in the range of pM), it even supports synapses by increasing synaptic strength in the hippocampus (Puzzo et al., 2008, 2011).

In addition to secretases, APP may be processed by caspases (in particular caspase-3), especially in cells undergoing apoptosis (Gervais et al., 1999; Pellegrini et al., 1999; Fiorelli et al., 2013). In neurons, caspase-induced processing of APP generates a C-terminal fragment (C31) with neurotoxic potential (Lu et al., 2003; Nguyen et al., 2008). Moreover, Fiorelli et al. (2013) showed that caspases cleavage generates two APP fragments (APP-Fs) of 25–35 kDa that are recognized by anti-Aβ antibodies.

Among the genetic risk factors for AD, the carriage of *APOE-ε4* allele plays a major role. Apolipoproteins carry lipids in the circulation and regulate lipid metabolism. ApoE, expressed predominantly in astrocytes, is suggested to be involved in redistribution of cholesterol and phospholipids during membrane remodeling (Holtzman et al., 2012). Links between AD and *APOE-ε4* are multiple: ApoE protein exists in 4 isoforms (E1 to E4), with ApoE3 being the most common allele, and it seems to play a role in Aβ fibrillogenesis and oligomerization as well as in Aβ clearance. Differently from the other three variants, ApoE4 exhibits scarce ability to bind Aβ, thus its expression contributes to Aβ accumulation and aggregation inside neurons and influences the formation of the parenchymal amyloid plaques (Holtzman et al., 2012; Verghese et al., 2013). When human ApoE isoforms were expressed in APP transgenic mice, differences in extracellular Aβ accumulation were observed in an isoform-dependent manner (E2 < E3 < E4; Holtzman et al., 2000). Similar results were also recently obtained by Hashimoto et al. (2012) who reported that the levels of Aβ oligomers in APOE ε4/ε4 AD brains were 2.7 times higher than those found in APOE ε3/ε3 AD brains, and that ApoE increased Aβ oligomer levels in an isoform-dependent manner (E2 < E3 < E4).

Finally, alterations of intracellular Ca^2+^ homeostasis and signaling have also been implicated in AD pathogenesis. It is well known that intracellular Ca^2^^+^, besides playing a pivotal role in a large number neuronal functions, is a critical determinant of neuronal survival and death (Piacentini et al., 2008a, b; Maiti et al., 2011; Bading, 2013). Several reports demonstrated correlation between Ca^2^^+^ homeostasis dysregulation leading to increased intracellular Ca^2^^+^ levels and AD (Green, 2009). Interestingly, ApoE expression in neuronal and glial cells correlates with increased intracellular Ca^2^^+^ concentrations in an isoform-dependent manner (E2 < E3 < E4; Müller et al., 1998).

## HOW CAN HSV-1 BE INVOLVED IN AD?

Herpes simplex type 1 virus is a neurotropic double-stranded DNA virus that primarily infects epithelial cells of oral and nasal mucosa. Here virus undergoes lytic replication; the newly produced viral particles may enter sensory neurons and, by axonal transport, reach the trigeminal ganglion where usually establishes a latent infection. The virus undergoes periodic reactivation cycles in which the newly formed viral particles are transported back to the site of primary infection through the sensory neurons, causing the well-known clinical lesions (i.e., cold sores and blisters). However, the bipolar trigeminal ganglion neurons also project to the trigeminal nuclei located in the brainstem. From here, neurons project to the thalamus to finally reach the sensory cortex. This is the path through which the reactivated virus may reach the central nervous system (CNS), where it may cause acute neurological disorders like encephalitis [herpes simplex encephalitis (HSE)] or a mild, clinically asymptomatic, infection, or establish life-long latent infection (Kastrukoff et al., 1982; Lewandowski et al., 2002; references in Dobson et al., 2003). The weakening of immune system occurring during aging may favor this process. In addition to the neuronal route, HSV-1 may enter the CNS through the blood stream, as demonstrated by Burgos et al. (2002, 2003, 2005). Many experimental evidence, described below, suggest that accumulation of intracellular damage caused by repeated cycles of viral reactivation may concur to neurodegeneration.

Some reports suggest that during infection herpes virus interacts with several human proteins that it uses to enter the cell and to move from plasma membrane to the nucleus and back (reviewed in Carter, 2008). HSV-1 also uses the host’s transcriptional machinery to replicate and binds to proteins that control immune surveillance or apoptosis. Noteworthy, in the attempt to eliminate the virus, host may even cause cell damage via immune and inflammatory responses targeting the virus-containing cells. If this happens in the CNS, HSV-1-induced inflammatory response may result in HSE or, in milder cases, in cell death and neurodegeneration.

Several epidemiological, immunological and molecular evidence link HSV-1 infections to AD pathogenesis. HSV-1 is a ubiquitous virus that affects more than 80% of people over 65 worldwide. The first evidence suggesting the involvement of HSV-1 in AD dates back to 1982 and is based on the observation that people surviving to HSE showed clinical signs reminiscent of AD (i.e., memory loss and cognitive impairment), and that brain regions primarily affected in HSE (limbic system, frontal and temporal cortices) were the same regions compromised in AD (Ball, 1982). During the last 30 years several research groups have conducted many studies providing solid support to the involvement of HSV-1 infection in AD pathogenesis. Here we will briefly summarize the main results of these researches.

### EPIDEMIOLOGICAL DATA

Several studies have been performed to assess the presence of HSV-1 in the brain of AD patients. After the first observations made by Ball (1982), other studies have demonstrated that HSE affected the same brain areas involved in AD in humans as well as in rodent experimental models (Damasio and Van Hoesen, 1985; Caparros-Lefebvre et al., 1996; Beffert et al., 1998; Wu et al., 2003; Taylor et al., 2007; Ando et al., 2008). Jamieson et al. (1992) and Itzhaki et al. (1997) reported that a high proportion (about 60%) of brains of elderly people contained latent HSV-1 DNA, especially in the CNS regions critically involved in AD. When present in AD brains, HSV-1 DNA was primarily located within amyloid plaques (Wozniak et al., 2009a). Whether or not the virus contributes to activation of pro-neurodegenerative pathways may largely depend on several host factors, including genetic predisposition, as described in greater detail below. Many infectious agents whose pathogenetic role has been established in other CNS diseases (e.g., varicella zoster virus, causing meningitis or encephalitis; Epstein–Barr virus, associated to both multiple sclerosis and CNS lymphoma; human herpes virus 6, associated to seizure in children) may infect subjects without producing evident clinical signs. On the other hand, even in the presence of “clinically silent” infection, virus may periodically reactivate and replicate in the CNS (Peter and Sevall, 2001; Bearer, 2012). There is no clear evidence on whether HSV-1 reaching the brain and infecting neurons resides there in latent form. However, the detection of HSV-1 DNA in the cerebro-spinal fluid (Deatly et al., 1988; Peter and Sevall, 2001; Plentz et al., 2008) suggests that HSV-1 replicates in the CNS (Bearer, 2012). The presence of HSV-1 proteins in hippocampal neurons of mice infected intraperitoneally with HSV-1 was demonstrated by Burgos et al. (2006a) who also showed that virus is reactivated by hyperthermia. After every reactivation, the newly produced viral particles generated by this “silent” replication and potentially reaching the brain might act, in a drop by drop fashion, to produce local brain damages that may largely differ from those of acute diseases like HSE. It has been recently reported that in brain of infected but asymptomatic mice, HSV-1 reactivation was associated to neuroinflammation and to the appearance of several markers of neurodegeneration including Tau hyperphosphorylation (Martin et al., 2013).

### IMMUNOLOGICAL DATA

A number of studies have been conducted to demonstrate the association between AD and HSV-1 infection by searching for antibodies against HSV-1 in the blood of AD patients. These studies revealed a strong correlation between AD occurrence and HSV infection or reactivation, as addressed in a longitudinal study including 512 elderly persons looking for correlation between anti-HSV-1 IgM positivity (a marker of virus primary infection or its reactivation) and development of AD-like cognitive dysfunctions (Letenneur et al., 2008). In the same study, no correlation was found between anti-HSV-1 IgG positivity and early dementia, thus suggesting that recurrent infection, rather the primary one, is dangerous for the CNS. Anti-HSV IgM levels, and not those of IgG, have also been found to inversely correlate with lower plasma Aβ levels (Féart et al., 2011). It is believed that increased amyloid deposition in the brain is paralleled by a lowering of Aβ levels in plasma and that low plasma Aβ levels might be considered possible short-term risk marker of dementia (Schupf et al., 2008; Lambert et al., 2009; Blennow et al., 2010). Another study recently demonstrated that HSV-1 reactivation, assessed as anti-HSV IgG avidity index (that is a more accurate way to demonstrate viral reactivation) occurs in prodromal AD and highly correlates with symptoms of mild cognitive impairment (Kobayashi et al., 2013). Finally, Mancuso et al. (2014) recently found that elevated HSV-1 antibody titers were significantly more frequent in AD patients than in control healthy patients and that they positively correlated with cortical bilateral temporal and orbitofrontal gray matter volume, that may be considered an index of AD pathology (Whitwell et al., 2012).

### GENETIC DETERMINANTS

Some studies have suggested that in people carrying the *APOE-ε4* allele and, therefore, predisposed to develop AD, HSV-1 infection markedly increases the risk of AD (Itzhaki et al., 1997; Itzhaki and Lin, 1998; references in Bearer, 2012). However, this correlation was not always confirmed (Beffert et al., 1998). APOE seems to affects the outcome of several different infections (Itzhaki et al., 1998; Dobson et al., 2003) and, interestingly, APOE-ε4 is a risk factor for cold sores (Itzhaki and Wozniak, 2008). Some studies also demonstrated that ApoE4 presence influences the viral load in the brain. Indeed, viral spreading into the brain of ApoE KO mice was lower than that occurring in wild-type mice and correlation was reported between ApoE expression and HSV-1 DNA concentration detected in the CNS (Burgos et al., 2002). In a subsequent study, the same authors showed that during acute infection with HSV-1, ApoE4 was more efficient than ApoE3 in promoting viral colonization of the brain (Burgos et al., 2006b). However, many genes and proteins implicated in AD, other than ApoE, have been found to interact with herpes simplex viral genome or regulate its life cycle, further supporting the hypothesized synergy between host and pathogens in causing AD-like brain damage (Carter, 2008). From this point of view, genome wide association investigations (GWAs) carried out in a large cohort of AD and non-AD subjects also suggested an infective etiology for sporadic AD. In particular, the polymorphism association of genes located on the chromosome 19 (i.e., Nectin-2 [NC-2]; APOE, glycoprotein carcinoembryonic antigen related cell adhesion molecule-16 [CEACAM-16]; B-cell lymphoma-3 [Bcl-3]; translocase of outer mitochondrial membrane 40 homolog [TOMM-40]) and on the chromosome 8 (complement receptor-1 [CR-l]; APOJ, C-type lectin domain A family-16 member [CLEC-16A]), results in a genetic fingerprint that might modulate brain susceptibility to herpes virus infection and lead to neuronal loss, inflammation and Aβ deposition (Porcellini et al., 2010; Licastro et al., 2011).

### MOLECULAR DATA

Several studies demonstrated that HSV-1 affects APP processing. HSV-1 infection may have profound effects on the host intracellular pathways leading to activation/inactivation of several signaling molecules and kinases involved in APP metabolism. Ruth Itzhaki’s group found that in human SH-SY5Y neuroblastoma cells HSV-1 induces APP cleavage with the production of a 55-kDa fragment (recognized in Western blot analysis by an anti-C-terminal APP antibody) starting from 6 h post infection (hpi), and the concomitant reduction of band intensity relative to full-length APP (Shipley et al., 2005). These authors also reported that HSV-1 infection caused Aβ40 and Aβ42 accumulation in human neuroblastoma and glioblastoma cells *in vitro*, whereas in brains of mice infected with HSV-1 they only found an increase in Aβ42 (Wozniak et al., 2007). However, further studies on *in vivo* models of recurrent HSV-1 brain infections are needed to determine the structural and functional alterations induced by viral reactivation.

**FIGURE 1:**
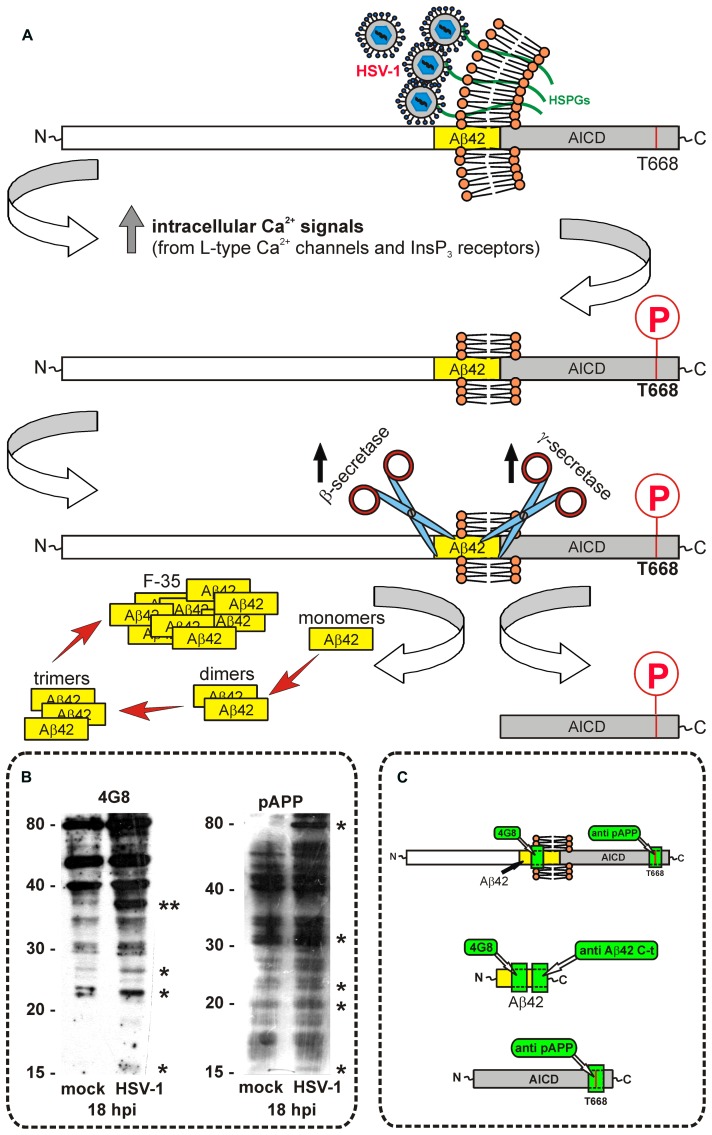
**HSV-1 infection induces APP cleavage**. **(A)** Our data suggest that HSV-1 binding to the heparan sulfate proteoaminoglycans (HSPGs) expressed on neuronal plasma membrane induces Ca^2^^+^ signals triggering APP phosphorylation at Thr668 that, in turn, increases β- and γ-secretase activity. As a result, there is an increased production of Aβ that aggregates to form oligomers of various size. The C-terminal APP fragment called AICD, created by the cleavage of γ secretase is phosphorylated at Thr668 and internalized in the nucleus where it may modulate gene transcription. **(B)** Western blots showing representative experiments performed with 4G8 antibody and anti pAPP^Thr668^ on intracellular lysates of neuronal cells. Asterisks in the blots indicate bands that are modified by 18 h of HSV-1 infection (hours post infection, hpi) with respect to the mock-infected conditions. Double asterisk in the Western blot for 4G8 indicates the APP fragment F35, characteristic of HSV-1 infected cells. **(C)** Cartoon indicating the amino acid sequences targeted by the different antibodies we used for Western blot and immunofluorescence experiments: 4G8 recognizes all APP fragments containing the amino acid sequence 17–24 of Aβ, independently on its cleavage. Therefore, it reacts with APP “full-length”, and with all APP fragments that contain Aβ 17–24 sequence; pAPP^Thr668^ antibody reacts with all C-terminus APP fragments containing phosphorylated Thr668, including APP full-length; anti Aβ42 C-terminus specifically reacts with the C-terminal part of Aβ42, and it does not recognize APP. Western blots in the panel **B** refer to previously published data (De Chiara et al., 2010).

**FIGURE 2:**
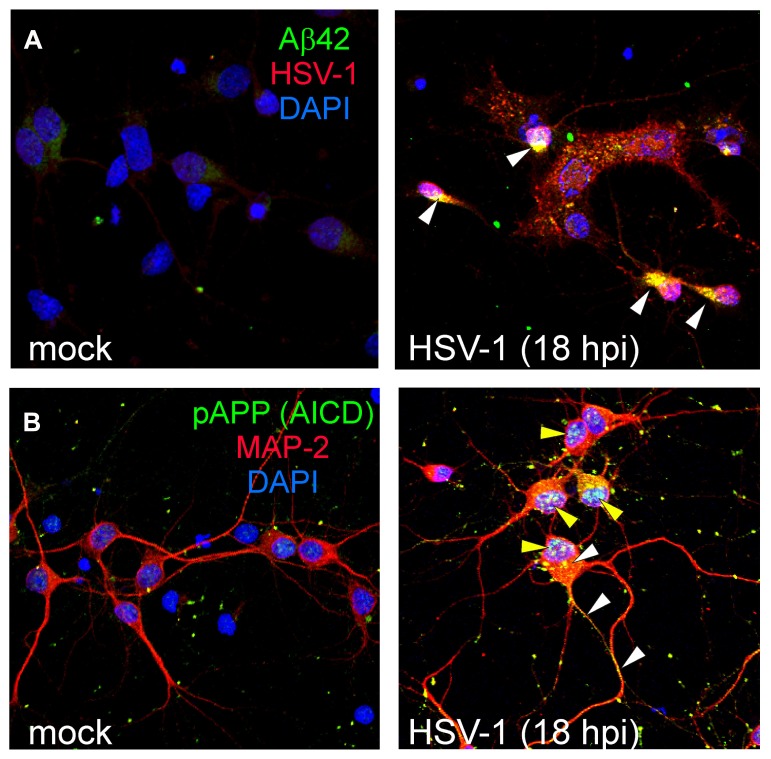
**HSV-1 infection induces intracellular accumulation of several APP fragments**. Representative examples of mock and HSV-1-infected (18 hpi) rat cortical neurons immunostained for Aβ42 C-terminus (green) and HSV-1 (red; **A)**; pAPP^Thr668^/AICD^pThr668^ (green) and MAP-2 (red; **B)**. Nuclei are stained in blue with DAPI. White arrowheads in the panel **A** (right) indicate intracellular accumulation of Aβ42 in infected (HSV-1-positive) neurons. In the panel **B** (right), yellow arrowheads indicate nuclear accumulation of AICD^pThr668^ in infected neurons (MAP-2-positive) whereas white arrowheads indicate staining for putative APP full-length phosphorylated at Thr668. Images in the panels **A** and **B** refer to previously published data (Piacentini et al., 2011).

We have demonstrated that in cultured neuronal cells HSV-1 induces amyloidogenic APP cleavage, with production of several APP-Fs including Aβ (De Chiara et al., 2010; Piacentini et al., 2011). In particular, we found that infection of SH-SY5Y human neuroblastoma cells and rat cortical neurons with HSV-1 induces multiple cleavages of APP, which result in the intra- and/or extra-cellular accumulation of several APP-Fs with neurotoxic potential. Among them we found: (i) APP-Fs of 35 and 45 kDa (APP-F35 and APP-F45) that comprise portions of Aβ; (ii) N-terminal APP-Fs that are secreted extracellularly; (iii) intracellular C-terminal APP-Fs, including AICD; and (iv) Aβ40 and Aβ42 in the form of monomers and small oligomers (dimers and trimers). Notably, the fragment APP-F35 seems to be a large Aβ oligomer (probably a nonamer) that is typical of viral-induced APP processing given that it was only found in cells infected with HSV-1. Indeed, it was revealed with antibodies recognizing amino-acidic sequences in Aβ (e.g., 4G8 and M2°, targeting amino acids 17–24 and 1–10 of Aβ, respectively), only 8–18 hpi (**Figures 1 and 2A**). Moreover, its formation was inhibited in the presence of β- and γ-secretase inhibitors. APP-F45 was revealed by M2° only. On the contrary, neither of these APP-Fs were recognized by antibodies raised against the N- and C-terminals of APP (**Figure 1**), thus indicating that they are not APP cleavage end-products. We also demonstrated that APP-Fs were present in the extracellular space and they had the ability to induce apoptosis in the neighboring cells (De Chiara et al., 2010). The multiple cleavages of APP occurring in infected cells are produced in part by known components of the amyloidogenic APP processing pathway, i.e., host-cell β-secretase, γ-secretase, and caspase-3-like enzymes, and in part by other cellular or viral enzymes not yet identified. Incidentally, the increased production of Aβ also reflected in an increased production and nuclear accumulation of AICD fragment that it is known to play a role in AD by modulating gene transcription.

It has been hypothesized that viral glycoprotein B is responsible for Aβ aggregation because this glycoprotein shares a significant portion of amino acid sequence similarity with Aβ, and it might serve as “core” to trigger Aβ aggregation (Cribbs et al., 2000). However, we and others (Wozniak et al., 2007; Piacentini et al., 2011) found that HSV-1-induced increased Aβ immunoreactivity was not due to cross-reactivity of the anti-Aβ antibodies with this glycoprotein.

From a functional point of view, we reported that HSV-1 binding to neuronal membrane induced membrane depolarization leading to increased neuronal excitability and triggering action potentials. This depolarization was due to activation of persistent Na^+^ currents and inhibition of leak K^+^ currents. Neuronal hyperexcitability persisted over time and was observed in neurons also at 12 hpi. Downstream to this effect we observed increased intracellular Ca^2^^+^ signaling, mainly due to activation of L-type Ca^2^^+^ channels and opening of inositol trisphosphate receptors (InsP_3_Rs) that caused marked intracellular Ca^2^^+^ entry from extracellular medium and Ca^2^^+^ release from intracellular stores (Piacentini et al., 2011). As above described, this Ca^2^^+^ dysregulation may trigger neurodegeneration (Mattson and Chan, 2003; Piacentini et al., 2008a, b; Chakroborty et al., 2012). We also found that HSV-1 induced Ca^2^^+^-mediated APP phosphorylation at Thr668. This is a key event critically involved in APP processing and Aβ formation (Pierrot et al., 2006). We demonstrated that HSV-1 infection induces a significant accumulation of Aβ42 inside neurons (**Figure 2**), and this effect depended on Ca^2^^+^ signaling activation. Indeed, in the presence of nifedipine and/or 2-aminoethoxydiphenyl borate (2-APB), that are specific blockers of L-type Ca^2^^+^ channels and InsP_3_ receptors, respectively, intraneuronal Aβ accumulation was significantly reduced (Piacentini et al., 2011). Besides accumulating inside neurons, Aβ monomers and small oligomers (dimers and trimers) were also released in the culture medium of infected neurons and revealed by Western blot analysis.

Several other studies have investigated the link between HSV-1 and AD at molecular level, independently on APP processing. It has been demonstrated that HSV-1 infection causes several functional and molecular alterations including: neurodegeneration and AD-like phosphorylation of tau protein (Zambrano et al., 2008; Wozniak et al., 2009b), the latter involving the activation of the glicogen sinthase kinase 3; caspase-3-mediated cleavage of Tau (Lerchundi et al., 2011); activation of the arachidonic acid cascade, which is involved in AD-type neuropathological changes (Hill et al., 2009). With regard to the interaction between herpes simplex viruses and APP, it has been recently shown that HSV-1 alters the distribution of cellular APP, and APP and HSV-1 capsid proteins physically interact to allow the migration of new viral particles inside infected cells by fast anterograde transport mechanisms (Cheng et al., 2011). This also suggests that HSV-1 affects APP dynamic and its intracellular distribution thus possibly causing alterations in its metabolism and processing.

## POSSIBLE HSV-1 RELATED THERAPEUTIC INTERVENTIONS

Experimental evidence reviewed above allows us to hypothesize that, in the near future, treatments aimed at preventing and/or delaying AD might include antiviral agents and/or target HSV-1-activated intracellular pathways. At the present, the main antiviral agent used for HSV-1 is Acyclovir. Acyclovir, that is the common name of acycloguanosine, targets infected cells and inhibits viral replication (Park et al., 1979). Wozniak et al. (2011) demonstrated that acyclovir reduced Aβ formation and Tau phosphorylation *in vitro*. This finding suggests that the appearance of molecular AD hallmarks depends on HSV-1 replication. Similarly, we observed that in rat cortical neurons, UV-inactivation of HSV-1 particles prevented Aβ42 formation and its intracellular accumulation, although the virus retained the ability to bind the neuronal membrane and trigger intracellular signaling leading to short-term APP phosphorylation (Piacentini et al., 2011). Itzhaki suggested that treatment with a variant of acyclovir, the valacyclovir, could be of great interest in treatment of HSV-1-induced neurodegeneration (Itzhaki and Wozniak, 2012). Indeed, valacyclovir displays greater bioavailability than acyclovir (5-fold to 10-fold), it crosses the blood brain barrier and has no toxicity when used in patients with multiple sclerosis (Friedman et al., 2005). Interestingly, lysine supplementation has been suggested to have a role in preventing the development of AD by reducing HSV-1 replication (Rubey, 2010).

Among the proposed pharmacological treatments for AD, the use of statins seems to be promising (Barone et al., 2013). It has been suggested that their beneficial effects may be related to their ability to modulate the entry of pathogens given that cholesterol synthesis inhibition blocks the entry, and limits neuronal spread, of HSV-1 (Hill et al., 2005).

Finally, some studies also suggested the use of GSK-3β inhibitors as possible candidates for AD treatment (King et al., 2014). Among them, lithium has been shown to have some beneficial effects on AD symptoms (see references in Forlenza et al., 2012; Nunes et al., 2013). Lithium has also been reported to inhibit HSV-1 replication both *in vitro* and *in vivo* (Ziaie and Kefalides, 1989; Ziaie et al., 1994; Amsterdam et al., 1996), thus allowing to speculate that its beneficial effects might also depend on its antiviral activity.

## CONCLUSION

Data reviewed here support the hypothesis that recurrent HSV-1 infection in the brain may have a critical role in AD pathogenesis by directly activating intracellular pathways leading to typical AD molecular hallmarks. Studies ongoing in our laboratory on *in vivo* models of recurrent mild brain HSV-1 infection will expectedly further support the causal relationship between HSV-1 reactivation in the CNS and AD-like cognitive decline. Collectively, data discussed in this manuscript indicate that greater attention should be paid to infectious and, especially, viral agents among the environmental factors contributing to AD pathogenesis.

## Conflict of Interest Statement

The authors declare that the research was conducted in the absence of any commercial or financial relationships that could be construed as a potential conflict of interest.
